# Endometrial scratching for poor responders based on the Bologna criteria in ICSI fresh embryo transfer cycles: a preliminary retrospective cohort study

**DOI:** 10.4274/jtgga.galenos.2021.2020.0127

**Published:** 2021-02-24

**Authors:** Şenol Kalyoncu, Aslıhan Yazıcıoğlu, Mustafa Demir

**Affiliations:** 1 Clinic of Obstetrics and Gynecology, TOBB ETÜ Faculty of Medicine Hospital, Ankara, Turkey; 2 Clinic of Obstetrics and Gynecology, Koru Hospital, Ankara, Turkey; 3 Clinic of Obstetrics and Gynecology, Anka Hospital, Gaziantep, Turkey

**Keywords:** Endometrial scratching, Bologna criteria, poor ovarian response, ICSI, GnRH antagonist protocol

## Abstract

**Objective:**

This study evaluated the effect of endometrial injury on pregnancy outcomes in patients with a poor ovarian response (POR), based on the Bologna criteria, who underwent intracytoplasmic sperm injection (ICSI) cycles.

**Material and Methods:**

Sixty-eight patients were enrolled in this retrospective cohort study. All patients in the endometrial scratching group (group 1, n=32) and control group (group 2, n=36) underwent office hysteroscopy in the early follicular phase of the cycle before controlled ovarian stimulation. Group 1 also underwent endometrial scratching. The main outcome measure was the ongoing pregnancy rate.

**Results:**

The study groups had similar baseline demographics, including age, body mass index, duration of infertility, number of ICSI cycles, and hormone levels. However, the antral follicle count was significantly higher in group 1 than in group 2 (4.2±1.9 vs 3.3±1.8; p<0.05). There were no significant group differences in ovarian stimulation characteristics (ovarian stimulation time, trigger day endometrial thickness, number of metaphase II oocytes), number of embryos transferred, or the ratio of embryo transfer on days 3 and 5. Moreover, there were no significant differences between groups 1 and 2 in the rates of chemical pregnancy (25% vs 19.4%), clinical pregnancy (15.6% vs 11.1%) or ongoing pregnancy (9.4% vs 8.3%) (p>0.05 for all).

**Conclusion:**

Endometrial scratching did not improve pregnancy outcomes for patients meeting the Bologna criteria for a POR to ICSI cycles using fresh embryo transfer and the GnRH antagonist protocol.

## Introduction

The world’s first in-vitro fertilization (IVF) baby was born in 1978. Since then, according to the International Committee for Monitoring Artificial Reproductive Technology, approximately 8 million babies have been born worldwide by means of IVF or other advanced infertility treatment methods. However, pregnancy rates in Europe appear to have stabilized, at approximately 36%, for both IVF and intracytoplasmic sperm injection (ICSI) treatment cycles (1).

The number of oocytes retrieved during an IVF cycle (ideally about 15) is directly related to the success thereof (2). A poor ovarian response (POR), in which controlled ovarian stimulation (COS) during IVF/ICSI cycles yields a limited number of oocytes, has an average prevalence of 6% to 35% (3,4). POR is frustrating for both patients and clinicians, as it is related to low pregnancy rates in IVF and high cancellation rates during COS (5). Thus, strategies to increase pregnancy rates in patients with POR are important in IVF/ICSI cycles.

Embryo implantation is one of the most important steps in the IVF cycle. Implantation failure may be caused by low endometrial receptivity, embryo problems, or abnormalities of both the endometrium and embryo. As reported previously, about 66% of implantation failures are caused by decreased endometrial receptivity (6). Thus, ensuring a receptive endometrium could increase pregnancy and delivery rates for artificial reproductive technology (ART). Various techniques have been used to ensure a receptive endometrium, including management of intracavitary abnormalities by hysteroscopy, treatment of thin endometrium, immunotherapy, and adjuvant treatments for women with POR (7,8).

Endometrial scratching, which is also called endometrial biopsy, endometrial injury, and endometrial trauma, has been offered by clinicians as a means of increasing endometrial receptivity, by an as yet unknown mechanism (9). Endometrial scratching can be done with endometrial biopsy instruments during the early follicular or luteal phase of the preceding IVF treatment cycle. The first study demonstrating a substantial increase in pregnancy rates after endometrial scratching was followed by many additional studies and reviews including various populations, although these have had conflicting results (10,11,12,13,14,15). However, no study has directly addressed the effect of endometrial scratching on patients with POR.

The objective of this preliminary trial was to assess the role of endometrial scratching in patients with a POR undergoing ICSI cycles with fresh embryo transfer.

## Material and Methods

This study included 68 women seen at a private IVF Center in Koru Hospital, Ankara, Turkey. Approval was obtained from the Koru Hospital Ethical Committee before the study commenced (approval number: 81, date: 04/11/2019). The study population was enrolled between January 1, 2017 and November 1, 2019. The Declaration of Helsinki was followed.

All participants were undergoing their first or repeated ICSI fresh embryo transfer and met the following inclusion criteria: fulfilment of the Bologna criteria for POR (16); aged 20-42 years; body mass index (BMI) 20-30 kg/m2; and a normal uterine cavity based on office hysteroscopy or hysterosalpingography. Patients were excluded if they had uterine anomalies, endocrine disorders, ovarian cysts, hydrosalpinx, or severe male factor infertility (e.g. aspermia, azoospermia), or if they had undergone uterine surgery in the last 3 months, or IVF cycles for preimplantation genetic diagnosis. Patients with genetic abnormalities, and those who had undergone cryo-thawed embryo transfer, were also excluded.

A detailed history, including age, previous treatments, and duration of infertility, was taken from all patients. A gynecologic examination consisting of a bimanual pelvic examination plus transvaginal ultrasonography (TVUSG) was conducted to check for structural abnormalities of the pelvis and ovaries. On days 2-5 of the menstrual cycle, gonadotropic hormone and estradiol concentrations were assessed.

All patients underwent office hysteroscopy during the early follicular phase of the menstrual cycle, i.e., immediately preceding the planned IVF cycle. A rigid 30°, 5.5 mm hysteroscope was used to perform hysteroscopy without anesthesia (Karl Storz Endoscopy, Tuttlingen, Germany). Serum physiological solution was used to distend the uterine cavity during hysteroscopy. Group 1, but not group 2, also underwent endometrial scratching during hysteroscopy; scissors were used to create mechanical tissue damage in local areas of the fundus, and in posterior and anterior regions of the endometrium.

COS began on day 2 or 3 of the menstrual cycle. Subcutaneous injections of recombinant follicle-stimulating hormone (FSH) (Gonal-F; Serono, Rome, Italy) or highly purified human menopausal gonadotropin (Menopur, Ferring, Sweden or Merional; IBSA, Collina d’Oro, Switzerland) were used for ovarian stimulation. The initial dose for each patient was based on the predicted ovarian response, and varied from 300 to 450 IU. On day 5 of COS, the flexible GnRH antagonist treatment protocol was implemented to prevent premature luteinizing hormone (LH) surge (Cetrotide; Merck Sharp and Dohme Ltd., Athens, Greece). Hormones, including FSH, estradiol, and thyroid- stimulating hormone, were measured before stimulation. LH, estradiol, and progesterone were also measured on the day of human chorionic gonadotropin (hCG) administration.

When two or more follicles with a diameter of at least 17 mm were detected, ovulation was triggered by a subcutaneous injection of 250 µgr r-hCG (Ovitrelle; Serono). Oocyte retrieval was carried out 35.5-36 hours after the r-hCG injection using TVUSG. On day 3 or day 5 after oocyte retrieval, depending on oocyte development, a maximum of two good-quality embryos were transferred into the uterine cavity using a Wallace semirigid catheter (Cooper Surgical, Malov, Denmark) under abdominal ultrasonography guidance.

Luteal support was provided until the 12^th^ week of gestation using Crinone gel (8% progesterone Serono). A pregnancy test was conducted approximately 2 weeks after embryo transfer. Clinical pregnancy was defined as any intrauterine gestational sac with a fetal heartbeat at 4 weeks after the first pregnancy test. The existence of at least one live fetus at the 12^th^ week of gestation was considered an ongoing pregnancy, which was the primary outcome measure.

### Statistical analysis

Statistical analysis was carried out using SPSS software (version 23.0; SPSS Inc., Chicago, IL, USA). Continuous variables are expressed as mean ± standard deviation or median (minimum-maximum). Categorical variables are expressed as number and percentage (%). The Kolmogorov-Smirnov test was used to check the distribution of the data. The Independent Samples t-test and Mann-Whitney U test were used to compare continuous variables. Pearson’s chi-squared test or Fisher’s exact test was used to compare categorical variables. A two-tailed p-value of <0.05 was considered significant.

## Results

A total of 68 patients were included in the present study. The endometrial scratching group (group 1) and control group (group 2) included 32 and 36 women, respectively. The groups were well-balanced in terms of baseline demographic data, including age, BMI, length of infertility, number of IVF cycles, and hormone levels (Table 1). However, the antral follicle count was significantly higher in group 1 compared to group 2 (4.2±1.9 vs 3.3±1.8; p<0.05).

Table 2 shows the COS, ICSI, and pregnancy outcomes. Groups 1 and 2 did not differ in duration of COS, trigger day endometrial thickness, or number of metaphase II oocytes retrieved. In addition, the number of embryos transferred, and the ratio of day 3 to day 5 embryo transfers, were similar between the two groups. Moreover, regarding pregnancy outcomes, groups 1 and 2 had similar rates of chemical pregnancy (25% and 19.4%, respectively), clinical pregnancy (15.6% and 11.1%) and ongoing pregnancy (9.4% and 8.3%).

## Discussion

This retrospective cohort trial investigated the impact of endometrial scratching on the pregnancy outcomes of women meeting the Bologna criteria for POR, who were undergoing ICSI fresh embryo transfer cycles using the GnRH antagonist protocol. The results showed that endometrial scratching did not increase rates of clinical or ongoing pregnancy.

Poor responders constitute a major challenge for ART. These patients are more likely to show poor oocyte retrieval and less favorable pregnancy outcomes. A previous review reported that 47 studies used 41 different definitions of POR (17). To overcome this challenge, ESHRE used the Bologna criteria to clearly define POR (16).

Endometrial injury may be useful in patients undergoing ART cycles, to improve endometrial receptivity and the chance of pregnancy. Although endometrial injury has been studied for over a decade, the biological mechanism by which it increases the chance of pregnancy is not clear. However, one putative mechanism is stimulation of the production of cytokines and growth factors, which are essential for endometrial receptivity and embryo implantation after endometrial scratching and subsequent healing (18).

Endometrial injury to improve the outcomes of IVF/ICSI cycles has attracted increasing attention since Granot et al. (19) conducted the first two studies in 2000 and 2003 (20). In the first study, repeated endometrial biopsies were performed to evaluate the endometrium of 12 infertile women with several unsuccessful cycles of IVF treatment. The authors noted that 11 of these women became pregnant during the first IVF cycle after endometrial biopsy (19). In the second study, a quasi-randomized prospective trial, the same authors reported that endometrial injury doubled the conception rate during IVF treatment (20).

Numerous subsequent studies have reported inconsistent results, including positive (21,22,23), negative (24,25), and neutral (26,27) effects of endometrial scratching in women undergoing IVF. Many reviews have also been published over the past 15 years. However, the conflicting results of randomized prospective studies are reflected in the lack of consensus in the interpretation thereof. Reviews articles have variously concluded that (i) endometrial scratching during ART cycles improves pregnancy outcomes (11,14,23); (ii) endometrial scratching during ART cycles does not improve pregnancy outcomes (12,13); and (iii) the evidence is insufficient for definitive conclusions to be drawn (23,28).

Previous trials of endometrial scratching during IVF cycles have included women with normal ovarian response (28), unselected populations (24,25,26,27), women undergoing their first IVF cycle (22), and women with one or more failed cycles (23,29,30). The present study is the first to recruit a subgroup of patients meeting the Bologna criteria for POR and undergoing an ICSI cycle with fresh embryo transfer. Our patients had similar pregnancy outcomes regardless of whether they underwent endometrial scratching during office hysteroscopy.

In most previous studies, endometrial scratching was done by endometrial aspiration using pipelle biopsy (22,23,24,25,26). Our study was consistent with previous ones in that scratching was completed during diagnostic hysteroscopy (28,29). The optimal time to induce endometrial injury is not clear; some propose that it should be performed during the luteal phase (22,24,25,28,29) while others suggest that it should be completed during the follicular phase (as in our study) so that there is more time for the effect of injury to become apparent (31). A previous head-to-head comparison found that proliferative-phase endometrial scratching conferred no advantage over endometrial injury during the luteal phase (32).

### Study Limitation

The retrospective nature of the present study is an important limitation. Another potential limitation is that endometrial scratching was performed during office hysteroscopy. The effect of hysteroscopy on endometrial receptivity could be similar to that of endometrial injury. However, we believe that  office hysteroscopy alone is insufficient for endometrial injury.

## Conclusion

The present study is the first to demonstrate that endometrial scratching does not improve pregnancy outcomes in patients meeting the Bologna criteria for POR, and undergoing ICSI cycles using fresh embryos and the GnRH antagonist protocol. Further randomized prospective trials using alternative endometrial scratching techniques and COS protocols, and larger samples drawn from this subgroup of patients, are needed.

## Figures and Tables

**Table 1:**
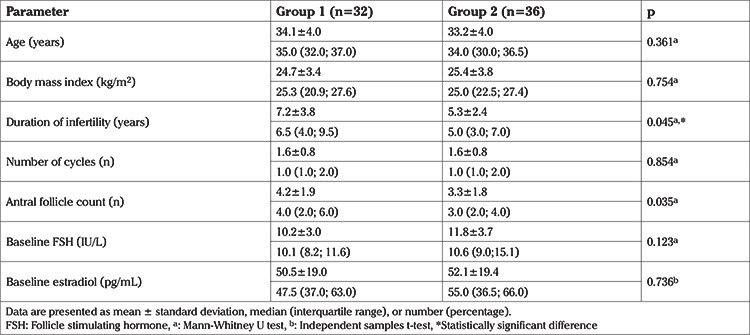
Baseline demographic, clinical, and laboratory characteristics of groups 1 and 2

**Table 2:**
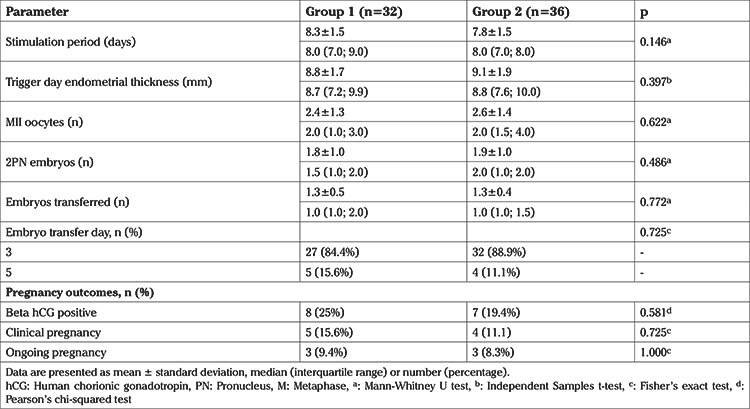
Controlled ovarian stimulation and pregnancy outcomes of groups 1 and 2
